# Editorial: Recent Advances in Diagnosis and Management of Urothelial Carcinoma

**DOI:** 10.3389/fonc.2021.656974

**Published:** 2021-03-29

**Authors:** Ja Hyeon Ku, Woonyoung Choi

**Affiliations:** ^1^ Department of Urology, College of Medicine, Seoul National University, Seoul, South Korea; ^2^ The Johns Hopkins Hospital, Johns Hopkins Medicine, Baltimore, MD, United States

**Keywords:** urothelial carcinoma, bladder cancer, upper tract urothelial carcinoma, diagnosis, management

## Introduction

Bladder cancer (BC) is the fourth most common cancer in men in the United States. The American Cancer Society estimates that 80,470 new cases of BC will be diagnosed in the U.S. in 2019 and that 17,670 people will die of the disease (1). Upper urinary tract urothelial carcinoma (UC) is a rare urologic malignancy, accounting for only 5% to 10% of all UCs (2); however, it shows an aggressive nature with high recurrence and progression rates (3). UC remains a common malignancy with few treatment advances in the last 20 years despite its high prevalence.

This Research Topic aims to report on, but is not limited to, the latest results of current and emerging diagnostic and therapeutic modalities used in the treatment of UC as well as basic science.

Liu et al. explored the potential functions and mechanisms of miR-221 in the autophagy and tumorigenesis of BC. They demonstrated that the downregulation of miR-221 and HMGA1 mediates autophagy in BC, and both of them are valuable therapeutic targets for BC.

Zhu et al. studied the role of activity-dependent neuroprotective protein (ADNP) in BC. The authors found that ADNP is overexpressed in BC and promotes cancer growth partly through AKT pathways. These authors argued that ADNP is crucial in predicting the outcome of BC patients and may be a potential therapeutic target in BC.

Mencucci et al. analyzed alterations in E-cadherin expression and EMT-related events in BC, and identified Ephrin-B1 as a new marker of tumor aggressiveness.

To predict immunotherapeutic biomarkers and identify new therapeutic targets, Cao et al. screened and identified immune-related cells and genes in the tumor microenvironment of BC based on the Cancer Genome Atlas (TCGA) Database and bioinformatics. They described that his study provides a new approach for immunotherapy researchers to explore immunotherapeutic cells and gene targets for the first time.

To date, there are no markers can replace or even reduce the use of routine diagnostic tools (cytology and cystoscopy). Elamin et al. assessed the changes in blood gene expression in BC patients and to identified genes serving as biomarkers for BC diagnosis and progression

Song et al. investigated new urine biomarkers for high-grade BC and investigated how they promote high-grade BC progression and thus affect the prognosis based on large-scale sequencing data. They indicated that ECM1, CRYAB, CGNL1, and GPX3 are potential urine biomarkers and could be served as new diagnostic and prognostic makers for high-grade BC.

Hayashi et al. developed urinary cell-free DNA (cfDNA) analysis by droplet digital PCR (ddPCR) as a high-throughput and rapid assay for BC detection and prognosis. They reported that ddPCR analysis of urinary cfDNA is a simple and promising assay for the clinical setting, surpassing UroVysion for detection and prognosis determination in BC.

Zhu et al. described the genetics-based molecular subtyping of BC. These authors claimed molecular subtyping provides more abundant tumor biological information and is expected to assist or replace the traditional classification system in the future.

Jung et al. investigated the prognostic implications of immunohistochemical staining for CK14 and the transcriptional characteristics associated with CK14 expression in papillary non-muscle-invasive upper tract UC. Authors indicated that CK14-positive cancer is an aggressive subtype with basal/squamous-like (BASQ) molecular characteristics and dynamic proliferative activity. Authors in the same institute analyzed the association of immunohistochemically defined BASQ and non-BASQ subtypes with two PD-L1 assays in muscle-invasive BC (Kim et al.). They revealed that a high PD-L1 positive rate was significantly associated with the BASQ-subtype.

To predict lymph node metastasis preoperatively in patients with T1 high-grade UC, Ou et al. developed and validated a nomogram. This nomogram incorporated the tumor number, tumor size, lymphovascular invasion, fibrinogen, and monocyte-to-lymphocyte ratio, which showed favorable predictive accuracy for lymph node metastasis.

Fuhrman et al. took a first look at real world data of checkpoint inhibitors for the treatment of metastatic UC after chemotherapy since the data on real world experience is scarce. Authors demonstrated promising overall survival and a low incidence for severe adverse events.

Yuk and Ku summarized the role of various systemic inflammatory responses (SIRs) in the treatment of patients with UC. Authors reviewed various SIRs related to UC, including C-reactive protein, albumin-globulin ratio, albumin, Glasgow prognostic score, modified Glasgow prognostic score, neutrophil-lymphocyte ratio, and platelet-lymphocyte ratio.

Two meta-analyses were included in this Research Topic. Using the method of systematic review and meta-analysis, Liu et al. showed that thermal intravesical chemotherapy can reduce the recurrence rate without increasing incidence of adverse events in patients with non-muscle invasive BC, when compared with normal temperature intravesical chemotherapy. Wu et al. investigated the potential prognostic role of serum lactate dehydrogenase (LDH) in patients with UC and indicated that high level of pretreatment serum LDH was associated with inferior outcomes in patients with UC.

This Research Topic described the current status of information relevant to UC in a variety of basic and clinical categories. Our hope is that this Research Topic contributes to improve outcomes for UC.

## Author Contributions 

JK and WC contributed to the writing and reviewing of this editorial. All authors contributed to the article and approved the submitted version.

## Conflict of Interest

The authors declare that the research was conducted in the absence of any commercial or financial relationships that could be construed as a potential conflict of interest.
